# Large Thermal Conductivity Differences between the Crystalline and Vitrified States of DMSO with Applications to Cryopreservation

**DOI:** 10.1371/journal.pone.0125862

**Published:** 2015-05-18

**Authors:** Lili E. Ehrlich, Justin S. G. Feig, Scott N. Schiffres, Jonathan A. Malen, Yoed Rabin

**Affiliations:** Department of Mechanical Engineering, Carnegie Mellon University, Pittsburgh, Pennsylvania, 15213, United States of America; University of California at Berkeley, UNITED STATES

## Abstract

Thermal conductivity of dimethyl-sulfoxide (DMSO) solution is measured in this study using a transient hot wire technique, where DMSO is a key ingredient in many cryoprotective agent (CPA) cocktails. Characterization of thermal properties of cryoprotective agents is essential to the analysis of cryopreservation processes, either when evaluating experimental data or for the design of new protocols. Also presented are reference measurements of thermal conductivity for pure water ice and glycerol. The thermal conductivity measurement setup is integrated into the experimentation stage of a scanning cryomacroscope apparatus, which facilitates the correlation of measured data with visualization of physical events. Thermal conductivity measurements were conducted for a DMSO concentration range of 2M and 10M, in a temperature range of -180°C and 25°C. Vitrified samples showed decreased thermal conductivity with decreasing temperature, while crystalline samples showed increased thermal conductivity with decreasing temperature. These different behaviors result in up to a tenfold difference in thermal conductivity at -180°C. Such dramatic differences can drastically impact heat transfer during cryopreservation and their quantification is therefore critical to cryobiology.

## Introduction

Cryopreservation is the preservation of biomaterials at low temperatures through suspension of mass transport. Ice crystallization is the cornerstone of cryoinjury, where cryopreservation—the preservation of biomaterials in cryogenic temperatures—revolves around the control of ice formation [1]. Ice formation is a path-dependent phenomenon, with the thermal history and availability of nucleators as dominating factors. Cryoprotective agents (CPAs) may be added to the cryopreserved biomaterial to suppress ice formation and growth during cryopreservation [2]. Ice-free cryopreservation can be achieved when high CPA concentration is loaded into the biomaterial and the material is cooled rapidly, in a process that is known as *vitrification* (*vitreous* means *glassy* in Latin) [3,4].

The physical property controlling the tendency to form glass is the CPA viscosity, which increases exponentially with the decreasing temperature, down to a temperature where the viscosity is so high that the material can be considered solid for all practical applications. This temperature is known as the glass transition temperature. Vitrification is achieved when the time required to cool the CPA is shorter than the typical time for the kinetic effect of crystallization. Hence, increasing the CPA concentration can decrease the likelihood of ice formation and promotes vitrification, with the adverse effect that the toxicity potential of CPA increases with the increasing concentration [5]. Alternatively, the cooling rate can be increased for lower-concentration CPA, with the adverse effect that rapid cooling can give rise to thermo-mechanical stress, eventually leading to structural damage. Balancing the competing needs to suppress crystallization, reduce toxicity, and preserve structural integrity, represents one of the major challenges in cryopreservation by vitrification of large-size specimens[6].

Investigation of cryopreservation in large-size specimens must rely on bioheat transfer simulations, as the path-dependent process can only be measured at discrete points, but the thermal history must be evaluated all across the specimen. These simulations necessitate knowledge of the thermal properties of the material, including thermal conductivity and specific heat. These properties may be strongly dependent upon temperature, solution concentration, and the level of molecular order—dependencies that too often are neglected, resulting in gross miscalculations of the temperature field [7]. For example, Choi and Bischof [8] have reviewed key thermal properties measurements and demonstrated how they may affect the outcome of thermal analysis as the process increasingly deviates from equilibrium conditions. Despite the need for specific thermal properties and in the absence of specific data, all too often thermal analyses rely on the properties of pure water (liquid and solid) as substitutes to CPA properties. In particular, the glassy state of CPA is often modeled as pure water ice or isotonic saline [9], despite their compositional and structural differences.

A hitherto overlooked consideration is the appreciable difference in thermal conductivity between an amorphous (i.e., vitrified) material and an ordered crystalline material of the same molecular composition. This difference results from long-range atomic periodicity in crystalline materials that enables efficient energy transport by collective motion of molecules (i.e., phonons) [10]. In contrast, uncorrelated atomic vibrations in amorphous solids transmit energy poorly. For example, the thermal conductivity of crystalline SiO_2_ (quartz) differs from amorphous SiO_2_ (glass) by one order of magnitude at 25°C and by as much as four orders of magnitude at -263°C [10].

This study focuses on thermal conductivity of dimethyl-sulfoxide (DMSO)—a glass-forming CPA—in its crystalline and vitrified states. This study also includes comparison of thermal conductivity of glycerol and pure water ice with literature data. Thermal conductivity measurements are paired with real-time observations of crystallization, vitrification, and fracture using a unique visualization device, known as the *cryomacroscope* [11]. This device allows uninterrupted visualization of the sample *in situ* during transient hot-wire measurements of thermal conductivity over a temperature range of -180°C and 25°C. DMSO is chosen as it is a relatively well characterized CPA [12–17], which serves as a key ingredient in many CPA cocktails. While the thermal conductivity of DMSO has been the focus of a related study [12], it has been limited to room temperature of -20°C or higher, to high concentrations of 7 M, 10.55 M, and 14 M (50%, 75%, and 100% by volume, respectively), where differentiation between the crystalline and the vitrified states obviously could not be made.

## Experimental Setup

**Fig 1:**
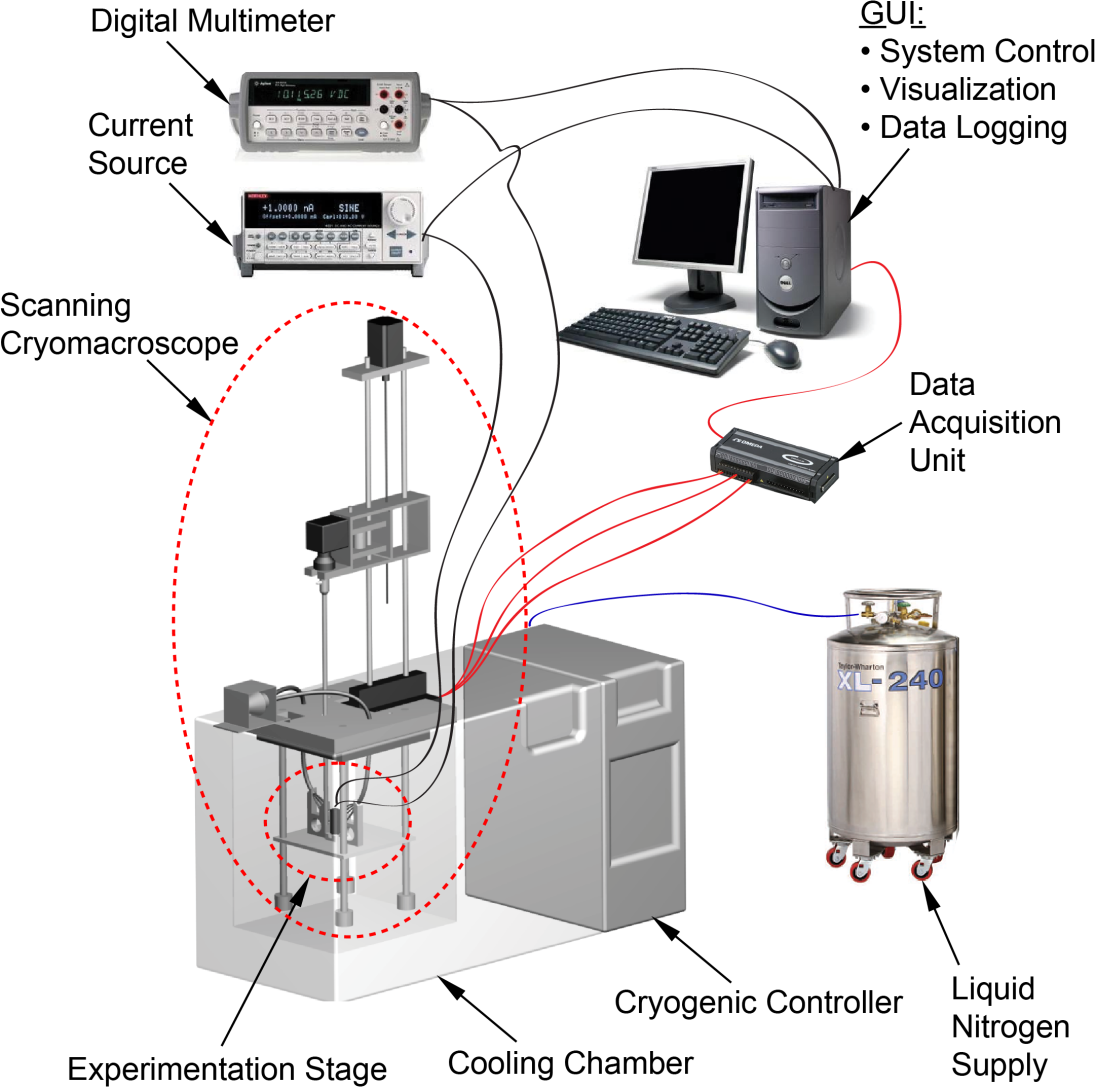
Schematic illustration of the scanning cryomacroscope setup and peripheral instrumentation [11]; the modified experimentation stage for thermal conductivity measurements is displayed with more detail in Fig 2.

**Fig 2:**
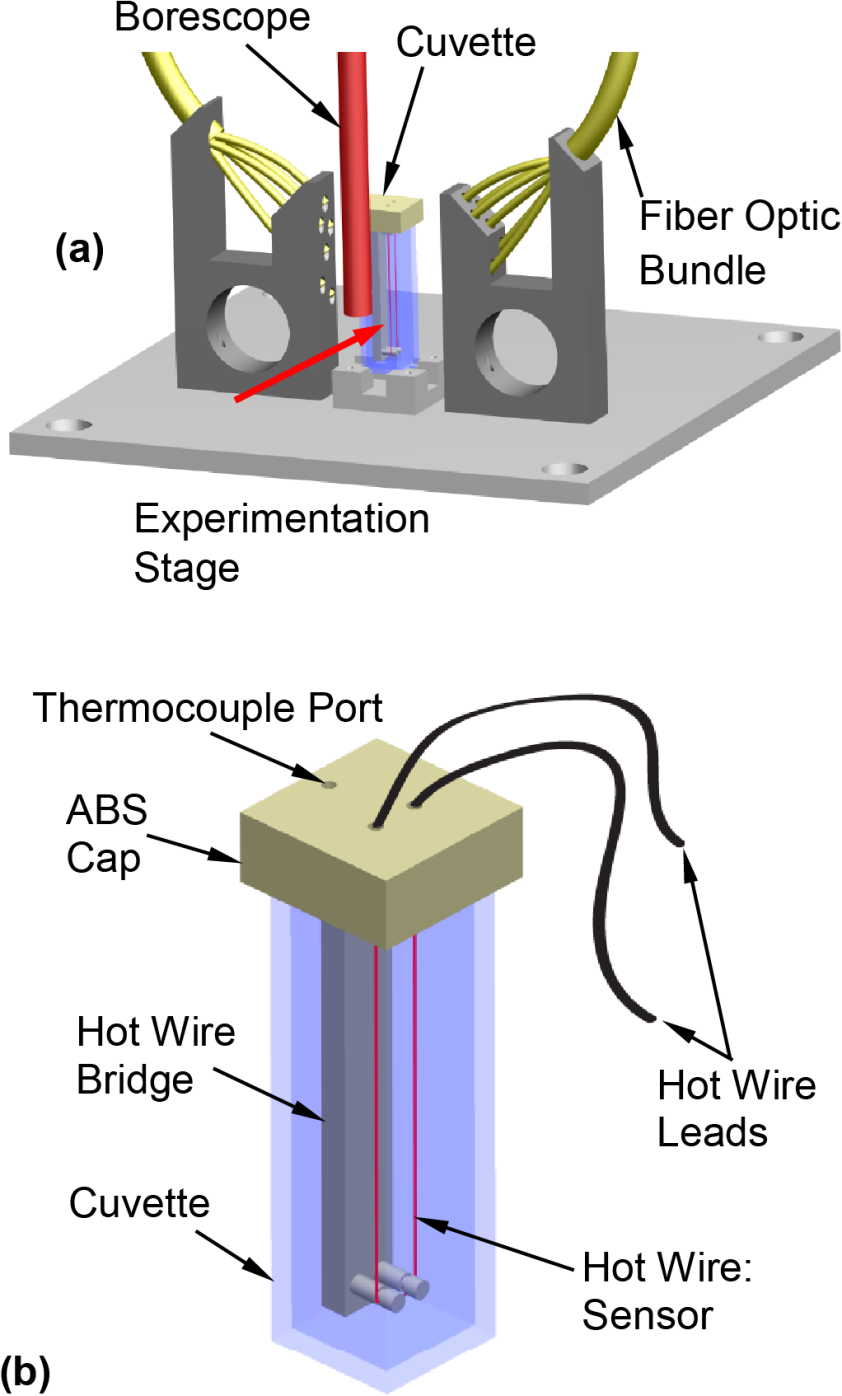
Schematic illustration of (a) the cryomacroscope experimentation stage (the red arrow represents the direction of view), and (b) the hot wire sensor setup in the cuvette (sample container).

The experimental setup is displayed in Fig 1, based on a previously developed visualization device for physical events during cryopreservation—the scanning cryomacroscope [11].While the scanning cryomacroscope has been presented in detail previously, it is presented here in brief for the completeness of presentation. Herein the unique instrumentation contribution to scanning cryomacroscopy is the development of an alternative experimentation stage for thermal conductivity measurements, as schematically illustrated in Fig 2. In general, the objective for cryomacroscopy is *in situ* investigation of the path-dependent process of cryopreservation. The scanning mechanism is integrated to enable the investigation of samples larger than the field of view of the optical component—the borescope.

The experimentation stage of the cryomacroscope is placed inside the cooling chamber of a top-loaded cooler, Kryo 10–16, controlled by a dedicated controller, Kryo 10–20 (Planer Ltd., UK). The controller can be programmed to cool and rewarm the chamber at rates up to -50°C/min and +10°C/min, respectively, in a temperature range of -180°C and room temperature. Temperature control is achieved by release of liquid nitrogen vapors into a circulated stream of heated air. This is a dual parameter control process, targeting both the rate of nitrogen supply and the power of the air heater.

The hot wire, which is the thermal conductivity sensor, is immersed in the sample during the entire cryogenic protocol, as displayed in Fig 2(B). The hot wire is held in place by means of a cap and bridge in one unit, which is 3D printed from ABS. The specific wire configuration was selected to ensure electrical resistivity values compatible with the peripheral instrumentation used: a current source (Model 6221, Keithley Instruments, Inc., Ohio) and a digital multimeter (Model 34401A, Keysight Technologies, Inc., Santa Rosa, CA). Detailed design considerations for the hot wire setup are separately described below.

Two means of temperature measurements are integrated into the system: thermocouples and the hot wire sensor itself. Three T-type thermocouples are strategically placed: (1) on the inner surface of the cuvette at its geometric center; (2) on the outer surface of the cuvette, opposing the first thermocouple; and, (3) in the free stream of air/nitrogen vapor mixture circulating through the chamber. Uncertainty in thermocouple measurements is estimated as ±0.5°C. Uncertainty in hot wire temperature measurements is estimated to be linearly dependent upon temperature, ranging from ±2.8°C at -178°C to ±0.5°C at 17.7°C, where the uncertainty analysis is presented in the S1 Appendix to this manuscript.

## Materials and Methods

This study focuses on measuring thermal conductivity of DMSO solution in the concentration range of 2M and 10M. In addition, pure water ice and pure glycerol are measured for reference, where water ice data [18] defines the upper boundary of thermal conductivity for DMSO solutions, and glycerol is an alternative glass-forming CPA [19,20]. While thermal conductivity data for glycerol are available in the relevant cryogenic temperature range, thermophysical properties of CPAs in general are only sparsely available in the literature.

In general, the critical cooling rate to ensure vitrification decreases with the increasing concentration. With the achievable cooling rates in the cooling chamber of the current system, 2M DMSO will always crystallize, 10M DMSO will always vitrify, and intermediate concentrations may either crystallize or vitrify to a variable degree, depending upon the concentration and the actual thermal history. Detailed analysis of the kinetics of crystallization in DMSO reveals a quite complex picture, which is beyond the scope of the current study. Being the first study of its kind and given the wide range of possibilities to design a thermal protocol in order to affect the path-dependent phenomenon of crystallization, a unified thermal protocol is selected for all experiments in the current study. The cooling rates in this thermal protocol are relevant to large-scale cryopreservation, which results in variable physical events along the cooling protocol for each DMSO concentration. Future studies are envisioned to study variable thermal protocols on specific DMSO concentrations, as well as expanding the base of knowledge to more complex CPA cocktails.

**Fig 3:**
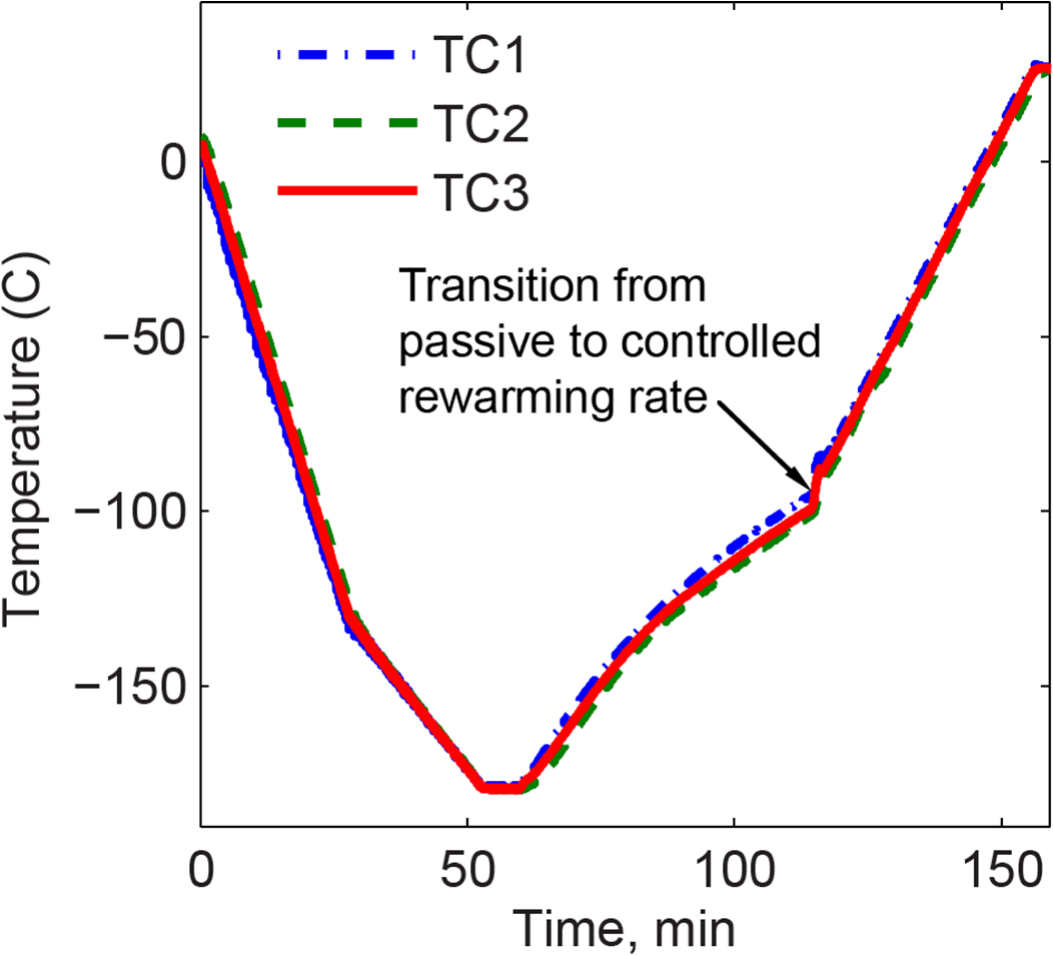
Typical thermal history during thermal conductivity measurements of 7.05M DMSO, where TC1 measures the chamber temperature, TC2 measures the wall inner surface at the center of one of the faces, and TC3 measures the temperature of the outer surface of the wall.

A typical thermal history for experimentation is displayed in Fig 3, which is comprised of: (1) precooling the chamber to about 10°C before the cryomacroscope is loaded on top of the cooling chamber, in order to reduce condensation on the system components (not shown in Fig 3); (2) cooling the sample at a rate of -5°C/min down to -130°C, then -2°C/min down to -180°C, with the reduced cooling rate below -130°C to avoid fracture formation as a result of thermo-mechanical stresses [21]; (3) passive rewarming up to -90°C, followed by a constant rewarming rate of +3°C/min back to room temperature. The passive rewarming phase at lower temperatures was required to eliminate temperature oscillations associated with cooling chamber control (inherent to the Kryo-16 and Kryo-20 systems).

### Hot-wire setup, design, and analysis

The transient hot-wire technique employs an immersed electrical resistor (a platinum wire) to simultaneously generate Joule heating and measure temperature (a resistance-based thermometer). In an ideal case, when the wire is immersed in an infinite domain (i.e., the CPA sample) and is subject to a step-like current activation, its transient thermal response can be best-fitted with experimental data to extract the thermal conductivity [22,23]. In practice, the parameters of the finite-volume sample, the sensor geometry, the electrical power generation, and peripheral sensing instrumentation must be carefully selected to closely approximate the ideal case, with the specific design considerations described below.

The hot-wire sensor in the current study is made from an approximately 70 mm-long platinum wire, 25.4 μm in diameter, having a 1.3 μm-thick isonel coating (A-M Systems, Sequim, WA, USA). A 4.5-mL polystyrene cuvette (Plastibrand) houses the sample fluid–sensor assembly as shown in Fig 2. The sensor wire is held in a U-shape configuration by a 3D printed cap and bridge in one unit (ABS). The length of the wire in this configuration yields a resistance in the range of 16 to 16.8 Ω, which is selected for its compatibility with the expected thermal conductivity values. In practice, each hot wire sensor used is calibrated for its specific reference values. Joule heating is generated by a constant current imposed for a period of 5 s, and is repeated in 35 s intervals during the entire rewarming phase of the thermal protocol (Fig 3). Simultaneously, voltage changes across the wire are measured at a frequency of 60 Hz.

For data analysis, the temperature elevation of the wire is given by:

$$\Delta T=\frac{\Delta R}{\beta R_{ref}}$$ (1)

where *β* is the measured coefficient of thermal resistance (*β* = 0.00411±0.00002°C^-1^for the eight sensors fabricated in the current study), *R*_ref_ is a reference resistance selected at 21°C, and the change in resistance Δ*R* is calculated as the ratio of the voltage change Δ*V* as a result of the applied current *I*:

$$\Delta R=\frac{\Delta V}{I}$$ (2)

The multimeter leads and the current source leads are hooked in parallel to both ends of the sensor wire in a four-point configuration, to eliminate parasitic lead and reduced contact resistance.

The method for extracting the thermal conductivity from the heated wire measurements has been published previously [22,23] and is summarized here in brief for the completeness of presentation. By rearranging the analytical solution for the corresponding transient line-heating problem, the thermal conductivity of the surrounding sample can be expressed as:

$$k_{sample}=\frac{q/4\pi }{d\left(\Delta T\right)/\;d\left(\ln t\right)}$$ (3)

where *∆T* is the temperature elevation in the wire, *t* is the elapsed time measured from the onset of heating, and *q* is the heat generation rate per unit length of wire:

$$q=\frac{I^{2}R_{0}}{L}$$ (4)

where *R*_0_ is the resistance of the wire at the onset of wire heating for the particular measurement, and *L* is the length of the platinum wire. Based on a detailed analysis of the solution provided by Nagasaka and Nagashima [23], the isonel coating does not influence the thermal conductivity measurements. This coating adds a constant shift to the transient temperature elevation, ∆*T*, while the surrounding sample affects the slope of the same curve, which is essentially used to extract *k*_sample._

**Fig 4:**
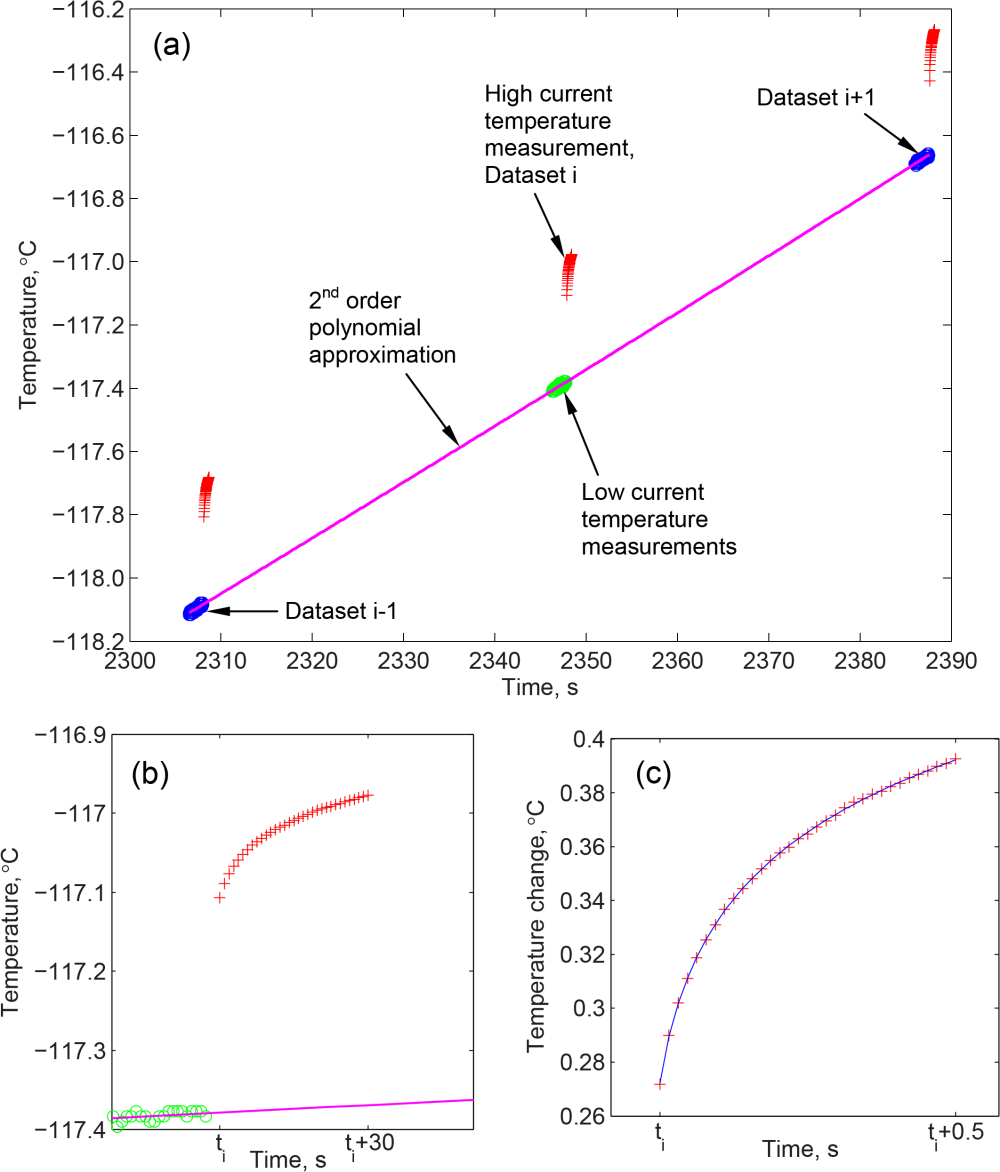
Temperature measurements during thermal conductivity experiments: (a) temperature results of three consecutive thermal experiments, where the change in the bulk sample temperature is best-fitted with a 2^nd^ order polynomial; (b) a higher magnification of an experimental dataset; and (c) a temperature dataset used to calculate the thermal conductivity after the subtraction of the bulk sample rewarming curve, where the slope of the best-fitted curve on a semi-log plot is used to calculate the thermal conductivity (shown as a solid line in figure).

Since temperature measurements with the wire sensor always generate Joule heating, special measures are taken to distinguish between temperature measurements before and during heating experiments. The experimental protocol combines two steps: (i) low–current resistance measurements in the range of 2 to 4 mA, to establish the preheating wire temperature (measured for 1.58 s at 60 Hz); and, (ii) high-current measurements in the range of 40 to 105 mA, based on the expected thermal conductivity, which defines a hot-wire experiment and marked with *t*_*i*_ in Fig 4 (duration of 0.5 s). Following the above analysis, the current applied during the low-current measurements is expected to elevate the wire temperature by the order of 10^–4^°C, which is considered negligible for the current analysis.

The solution presented in Eqs (1)–(4) has been developed under the assumption of an infinite domain, initially at a uniform temperature. However, thermal conductivity measurements in the current study are taken continually, as the finite sample rewarms. Modeling the hot wire response in the current experimental setup with the above solution is justified for the following reasons (see also Fig 4):

- the thermal penetration depth as a result of the step-like heat generation, *x*_*TP*_, is conservatively estimated to be shorter than the distance between the hot wire and any adjacent object, as further discussed below in the context of Eq (5);
- the ideal model errors due to axial heat loss are negligible given the radius and length of the wire sensor, the ratio of *k*_sample_ to *k*_wire_, and the ratio of ρ_sample_ *C*_p,sample_ to ρ_wire_ *C*_p,wire_ [24];
- the time interval between consecutive heating events (35 s) is long enough for the wire to return to its surroundings temperature;
- the heating rate of the wire during a single heating event (35°C/min on average), is an order of magnitude faster than the overall rewarming rate of the sample from cryogenic temperatures (about 3°C/min);
- the warming rate across the sample is uniform, causing the sample to rewarm as a thermally lumped system [7]; and,
- the thermal mass of the wire is four orders of magnitude smaller than that of the heated region in the sample.

Thermal modeling of the hot wire response to step-like heating can be done with Eqs (1)–(4) by decoupling it from the response of the bulk sample to cooling by the cooling chamber. These two processes can be decoupled since the heat diffusion equation is linear, which permits superposition of solutions [25]. The following procedure has been devised to capture the temperature of the bulk sample, as also illustrated in Fig 4. A second-order polynomial is fit to the low-current measurements taken before each high-current dataset (*t*_i-1_, *t*_i_, and *t*_i+1_ in Fig 4A). From each dataset, 95 consecutive measurements are used for the best-fit polynomial approximation, resulting in a total of 285 data points. Temperatures from the above polynomial approximation based on the low-current measurements are subtracted from the high-current measurements to evaluate Δ*T* as a result of the heating experiment. Finally, a linear curve is best-fitted for the rate of change of Δ*T* with respect to ln(*t*), which serves as the basis for the calculation of the thermal conductivity in Eq (3). An example of this fit is shown in Fig 4C.

In order to estimate the thermal penetration depth for the purpose of system design, a simplified solution for the temperature distribution in response to a sudden application of a constant heat flux is used [25]. This solution is given for a semi-infinite domain in a Cartesian geometry, while heat transfer around the heated wire is radial in nature. Hence, the solution from [25] serves as a conservative measure, where heat diffusion in a cylindrical system will make the actual penetration depth from the heated wire smaller and its decay faster. According to the above solution, the thermal penetration depth is given by:

$$x_{TP}=\sqrt{4\alpha t}$$ (5)

where *α* is the thermal diffusivity of the sample. In general, the thermal diffusivity increases with the decreasing DMSO concentration and with the decreasing temperature. For example, the thermal diffusivity of 2M DMSO at -180°C is 3.56×10^–6^ m^2^/s, and a heating duration of less than 0.73 s is required since the wire is placed 3.2 mm away from the cuvette wall. In practice, all DMSO experiments were performed for a heating duration of 0.5 s. Reference experiments on pure water ice were all performed for a heating duration of less than 0.35 s for similar considerations.

## Results and Discussion

**Fig 5:**
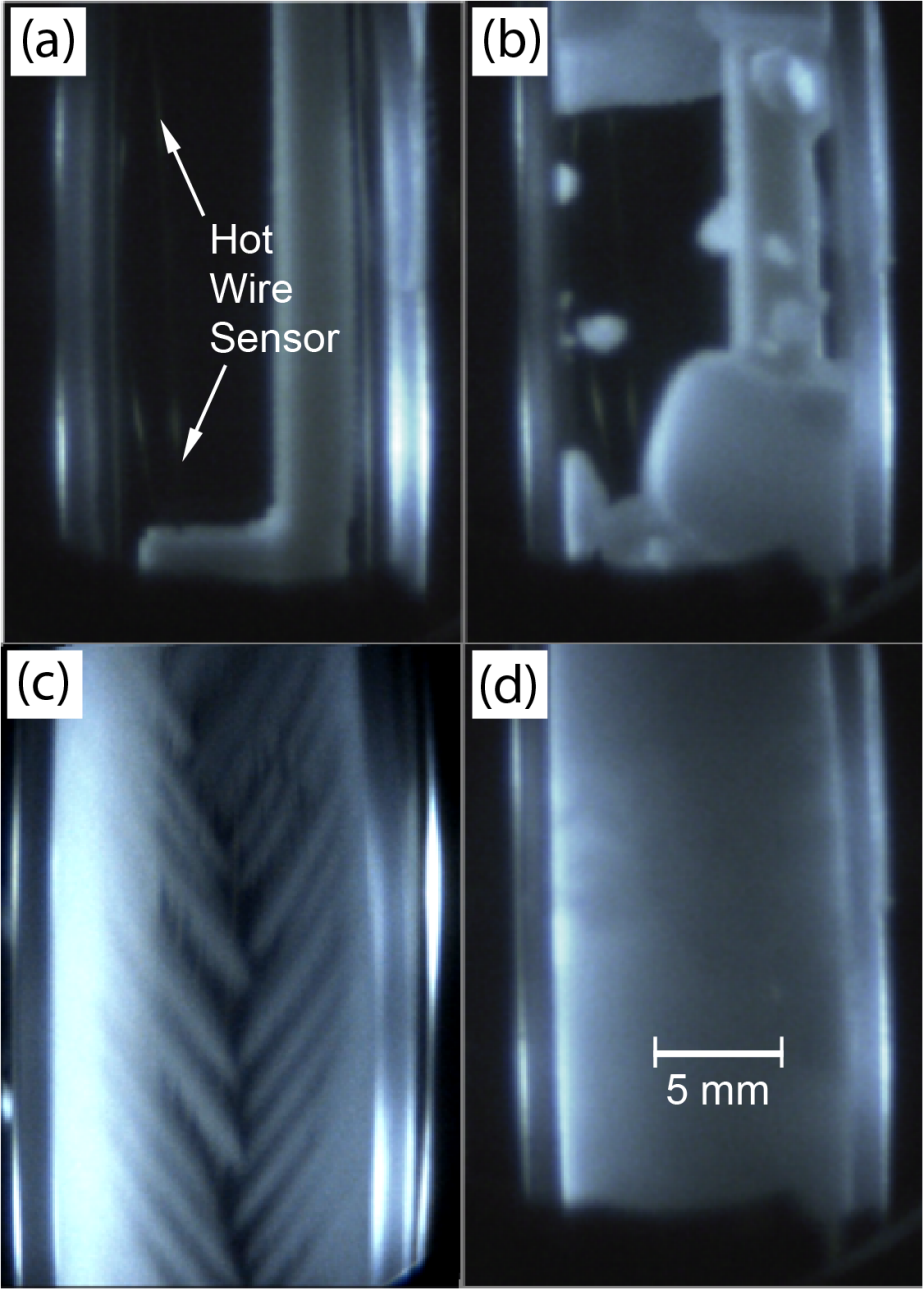
Cryomacroscope images of samples in various states: (a) a vitrified 7.05M DMSO sample at a temperature of -147°C; (b) a 2M DMSO sample undergoing crystallization in the form of dendrites at temperature of -10°C; (c) a partially crystallized 6M DMSO sample at a temperature of -58°C; and (d) a completely crystallized 6M DMSO solution at a temperature of -65°C.

Images of DMSO samples from the scanning cryomacroscope are shown in Fig 5. Complete vitrification in 7.05M DMSO is displayed in Fig 5(A) at an inner wall-surface temperature of -147°C, which is transparent in the glassy state. Also shown there is the wire sensor. The solution of 7.05M DMSO has the same overall molar concentration as of the CPA cocktail VS55, which has drawn significant attention in the cryobiology community in recent years. Both VS55 and 7.05M DMSO display similar mechanical behavior [26,27]. Crystal growth in finger-like formation (also known as *dendrites*) is displayed in Fig 5(B), for 2M DMSO at an inner wall-surface temperature of -10°C. Partial vitrification and complete crystallization in 6M DMSO are displayed in Fig 5 (C) and 5 (D), respectively.

Evidently, simultaneous observations of physical events during thermal conductivity measurements are essential for experimental data interpretation, where Fig 5 displays only selected scenarios out of a virtually endless spectrum of possibilities. For example, localized DMSO boiling occurred during preliminary testing in the current study, which effectively resulted in measuring the thermal conductivity of DMSO vapors, although the surrounding material was maintained in cryogenic temperatures. That problem of boiling was created by overpowering the hot wire sensor. While this report focuses primarily on thermal conductivity data, it has been routinely compared against simultaneous video recording of each experiment.

### Reference Experiments

The experimental apparatus and analysis technique were first evaluated against available data from the literature for relevant materials: pure water ice [7,28] and glycerol [19,20]. While glycerol is a known cryoprotective agent at various concentrations, comparable data in this temperature range is available only for pure glycerol[19,20].

**Fig 6:**
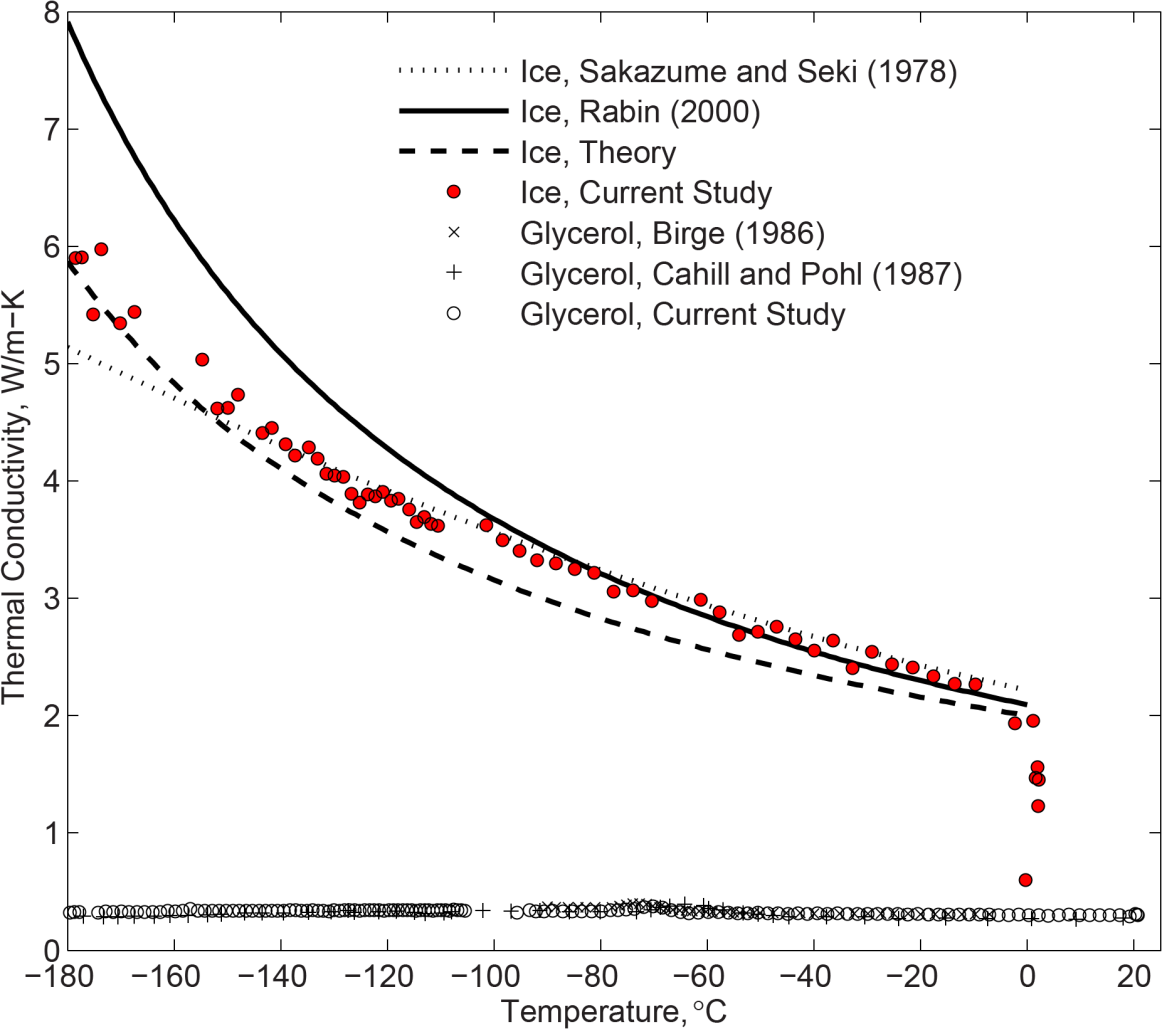
Thermal conductivity measurements of pure water ice and glycerol in the current study, compared with literature data, where the curve by Rabin (2000) [7] represents compilation of earlier literature data.

Theoretical studies suggest that the thermal conductivity of water ice within the relevant portion of the cryogenic temperature range should have the following functional behavior (Fig 6):

$$k=\frac{a}{T^{b}}$$ (6)

where *a* and *b* are constants and the temperature is measured in an absolute scale. For pure water ice, theoretical considerations suggest values of 546 W/m and 1 (dimensionless) for *a* and *b*, respectively [7]. Rabin compiled previously published data and found that, while the theoretical behavior follows experimental findings, best-fitting this functional behavior with experimental data leads to *a* and *b* values of 2135 and -1.235, respectively [7]. Sakazume and Seki [28] suggested a more moderate increase of thermal conductivity with the decreasing temperature. It can be seen from Fig 6 that the experimental data obtained in the current study follow closely the compilation by Rabin down to -100°C, Sakazume and Seki data down to -140°C, below which new the new experimental data lay in between those earlier publications.

In the amorphous state, the thermal conductivity of glycerol is expected to monotonically and moderately decrease with the decreasing temperature. It can be seen from Fig 6 that the current experimental data agrees very well with the previously published data, where the differences are generally within the estimated uncertainty in thermal conductivity measurements. It is concluded that the experimental setup and analysis technique are valid for thermal conductivity measurements, and the discussion now turns to new findings on DMSO.

### DMSO Experiments

**Fig 7:**
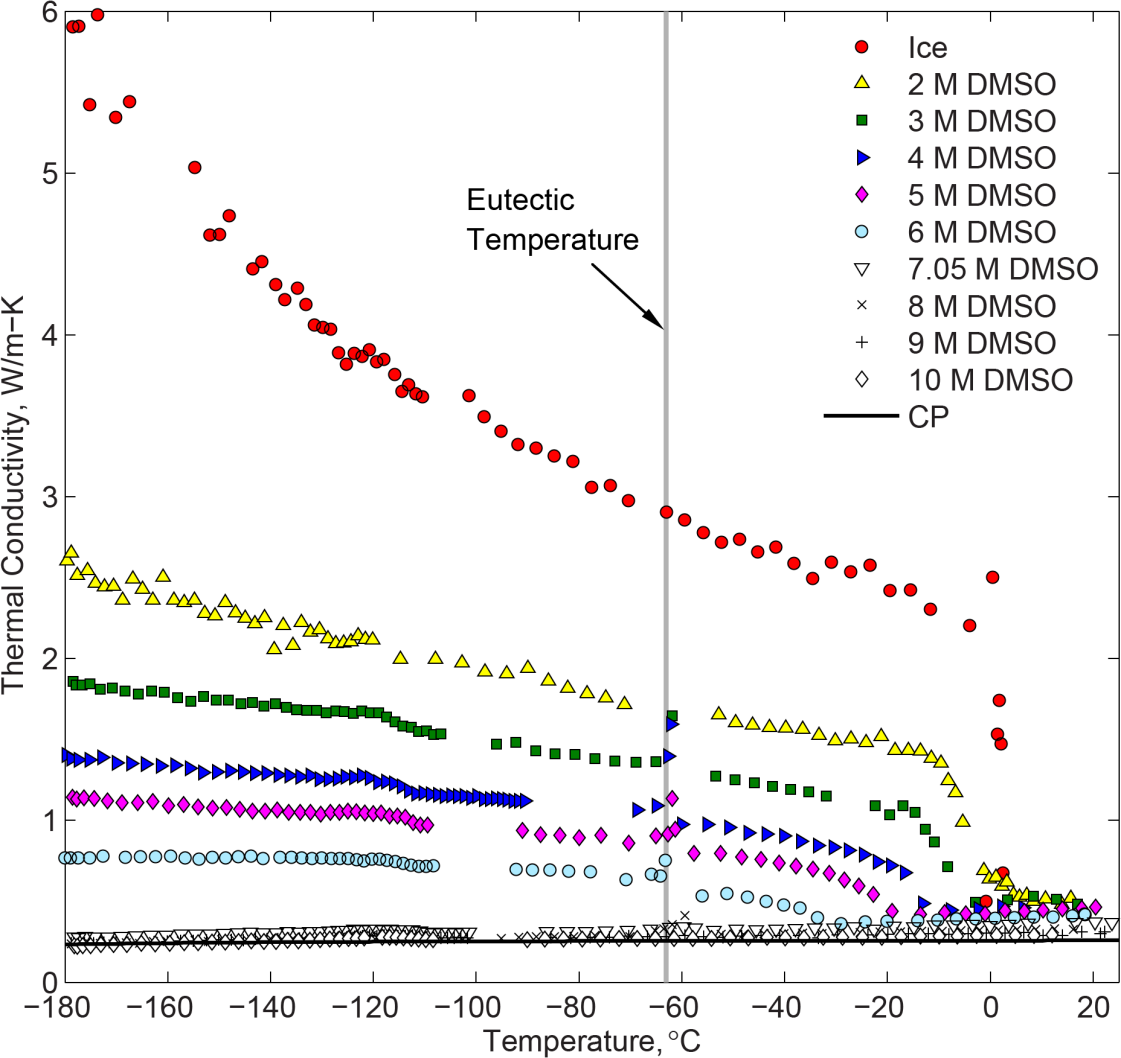
Thermal conductivity measurements of DMSO and pure water ice. The Cahill-Pohl model for thermal conductivity of amorphous solids is calculated with Eq (8) for 10 M DMSO. Based on cryomacroscope observations, DMSO concentrations of 6M or less underwent crystallization while concentrations of 7.05M and above vitrified.

Fig 7 displays experimental results for DMSO at various concentrations and for pure water ice for reference. A gap is present in each dataset around the time at which cooling chamber control is switched from passive to controlled heating. This change in control mode created temperature oscillations in the sample, which did not permit the data analysis technique discussed in conjunction with Fig 4. Best-fit coefficients for all the experimental results shown in Fig 7 are listed in Table 1 for the benefit of future cryobiology analyses.

**Table 1:** Best-fit polynomial approximation data for the thermal conductivity curves displayed in Fig 7.

DMSO Concentration	Temperature Range	Polynomial Approximation, °C	R^2^ value
2M	-180.0°C … -9.4°C	7.63×10^–10^ T^4^+1.60×10^–7^ T^3^+9.58×10^–6^ T^2^-6.01×10^-3^T+1.32	0.981
	-9.4°C … -0.2°C	1.41 ×10^–3^ T^2^-6.86 ×10^-2^T+6.06×10^–1^	0.991
3M	-180.0°C … -17.5°C	-1.55×10^–9^ T^4^-5.81×10^–7^ T^3^-8.01×10^–5^ T^2^-9.82×10^-3^T+9.00×10^–1^	0.996
	-17.5°C … -11.5°C	-3.97×10^–3^ T^2^-1.40×10^-1^T-1.84×10^–1^	*
	-11.5°C … -5.4°C	-3.47×10^–3^ T^2^-1.25×10^-1^T-7.92×10^–2^	*
	-5.4°C … +18.2°C	1.94 ×10^–3^ T+5.04 ×10^–1^	*
4M	-180.0°C … -24.2°C	-1.44×10^–9^ T^4^-6.42×10^–7^ T^3^-1.08×10^–4^ T^2^-1.16×10^-2^T+5.67×10^–1^	0.997
	-24.2°C … -17.3°C	-2.11×10^–3^ T^2^-1.06×10^–1^ T-5.35×10^–1^	*
	-17.3°C … -13.6°C	-1.97×10^–4^ T^3^-1.51×10^–2^ T^2^-3.89×10^-1^T-2.56	*
	-13.6°C … +8.4°C	-1.93×10^–3^ T+4.58×10^–1^	*
5M	-180.0°C … -32.7°C	-4.48×10^–10^ T^4^-2.76×10^–7^ T^3^-6.58×10^–5^ T^2^-1.16×10^-2^T+4.45×10^–1^	0.997
	-32.7°C … -19.2°C	-9.77×10^–4^ T^2^-7.15 ×10^-2^T-6.00×10^–1^	*
	-19.2°C … +20.6°C	1.23×10^–3^ T+4.34×10^–1^	0.916
6M	-180.0°C … -47.3°C	-6.65×10^–10^ T^4^-3.94×10^–7^ T^3^-1.01×10^–4^ T^2^-1.31×10^-2^T+9.31×10^–2^	0.971
	-47.3°C … -37.7°C	-3.85×10^–4^ T^2^-3.97×10^-2^T-4.91×10^–1^	*
	-37.7°C … -32.0°C	-6.27×10^–4^ T^2^-5.98×10^-2^T-9.06×10^–1^	*
	-32.0°C … +18.5°C	9.25×10^–4^ T+3.96×10^–1^	0.932
7.05M	-180.0°C … +25.5°C	-2.95×10^–10^ T^4^-6.87×10^–8^ T^3^-1.29×10^-6^T^2^+7.42×10^-4^T+3.56×10^–1^	0.982
8M	-180.0°C … +17.2°C	-2.41×10^–10^ T^4^-5.76×10^–8^ T^3^-2.31×10^-6^T^2^+5.57×10^-4^T+3.23×10^–1^	0.989
9M	-180.0°C … +22.3°C	-2.02×10^–10^ T^4^-4.57×10^–8^ T^3^-1.90×10^-6^T^2^+3.25×10^-4^T+3.01×10^–1^	0.948
10M	-180.0°C… +13.8°C	-1.14×10^–10^ T^4^-1.50×10^–8^ T^3^+1.07×10^-6^T^2^+3.74×10^-4^T+2.87×10^–1^	0.987

* A dataset consisting of fewer than ten data points

It can be seen from Fig 7 that the thermal conductivity of DMSO decreases with the increasing concentration. For DMSO concentration of 7.05M or higher, complete vitrification was observed while the thermal conductivity displayed a monotonic decrease in value with the decreasing temperature. In terms of thermal conductivity, the vitrified material appears to smoothly follow the trend from the liquid phase at higher temperatures. Crystallization was apparent in DMSO concentrations of 6M or less, which caused significant increase in the thermal conductivity as crystallization progressed. Thermal conductivity of DMSO follows opposing trends in the vitrified and crystallized phases, with the latter increasing with the decreased temperature.

### Crystallized DMSO

For samples that crystallized at lower temperatures, the thermal conductivity initially decreases with the decreasing temperature in the liquid phase, down to the onset of crystallization, and then dramatically increases as phase transition progresses. The temperature dependent behavior of thermal conductivity is consistent with other crystalline solids at temperatures above their peak in thermal conductivity. As crystals form in the sample, phonons become the dominant heat carriers. As temperature decreases further, high energy phonons become deactivated and the reduced phonon population leads to decreased phonon-phonon scattering, while increasing the mean free paths of the remaining phonons. Thermal conductivity continues to increase with the decreasing temperature within the studied cryogenic range, as the phonon mean free paths continue to increase. At lower temperatures, which are typically beyond the scope of cryobiology applications, one would expect the thermal conductivity to peak and then decrease with decreasing temperature as deactivation of phonons ultimately outweighs increases in the mean free paths of residual low energy phonons.

**Fig 8:**
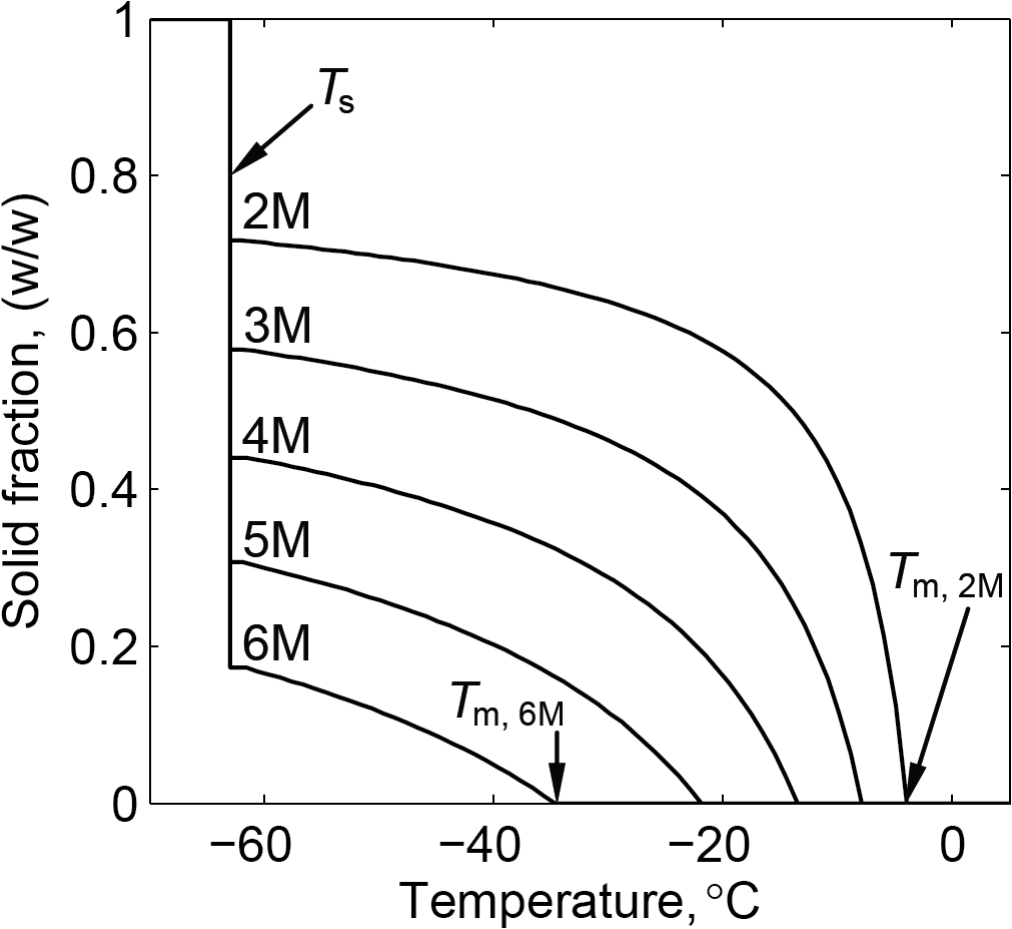
Solid fraction during solidification of a water-DMSO mixture, extracted from a phase diagram [13].

Fig 8 displays the solid fraction in the water-DMSO mixture at equilibrium, extracted from a phase diagram [13].Table 2 lists the liquidus temperature, *T*_*l*_, above which the material is completely liquid, also shown in Fig 8. Table 2 also lists the temperature at which melting completion is observed in the samples, *T*_*m*_, based on thermal conductivity measurements. The temperature *T*_*m*_ is estimated as the intersection point of two best-fitted curves: (i) a first-order polynomial approximation for the liquid phase above *T*_*l*_, and (ii) a second-order polynomial approximation for the closest five sampled points below *T*_*l*_. Listed uncertainty values for *T*_*l*_ in Table 2 is attributed only to the quality of data harvesting from the phase diagram [13], while uncertainty in the compilation of the phase diagram remains unknown. While a good agreement is displayed between *T*_*l*_ and *T*_*m*_ in Table 2, it should be noted that *T*_*l*_ corresponds to equilibrium conditions, while *T*_*m*_ corresponds to a process. Nevertheless, the close agreement between *T*_*l*_ and *T*_*m*_ suggests that the rewarming rate (Fig 5) can be considered very slow, and that the process can be first-order approximated as a quasi-steady.

**Table 2:** The temperature of melting completion based on experimental results, *T*_*m*_, observed during thermal conductivity measurements in comparison with the liquidus temperature, *T*_*l*_, from a water-DMSO phase diagram [9].

DMSO Concentration	*T*_*l*_, °C ^‡^	*T*_*m*_, °C ^†^
2M	-3.9 ± 0.9	*
3M	-7.9 ± 0.9	-5.4 ± 0.5
4M	-13.5 ± 1.1	-13.6 ± 0.5
5M	-21.9 ± 1.3	-19.2 ± 0.5
6M	-34.6 ± 1.4	-32.0 ± 0.5

* No definite observation could be made

‡ Uncertainty only due to extraction of data from the phase diagram in [9]

† Uncertainty only due to temperature measurements in the current study

The solidus temperature, *T*_*s*_, below which the material is completely solid, is -63±0.9°C for DMSO concentrations below 8M at equilibrium conditions (equal to the eutectic temperature found for 7.4M) [13]. Higher than expected thermal conductivity values were found around this temperature for DMSO concentration lower than 8M (highlighted with a gray line in Fig 8), which are non-physical artifacts, resulting from latent effects and the method of thermal conductivity interpretation (illustrated in Fig 4). In practice, experimental data suggests the onset of melting around the predicted value of -63°C with peak value between -62.3° and -60.1°C, for lower concentrations than 8M.

Within the temperature range one may assume lower concentration DMSO solutions as composed of water ice crystals suspended in molten and concentrated DMSO. A simple way of estimating the effective thermal conductivity of a randomly distributed two-component medium is proposed by the Bruggeman model [29]:

$$v_{1}\frac{\kappa_{1}-\kappa_{e}}{\kappa_{1}+\kappa k_{e}}+\left(1-v_{1}\right)\frac{\kappa_{2}-\kappa_{e}}{\kappa_{2}+2\kappa_{e}}=0$$ (7)

where is the volume fraction, *κ* is the thermal conductivity of the components, the indices 1 and 2 refer to different medium components, and the index *κ*_*e*_ is the effective thermal conductivity of the medium.

**Fig 9:**
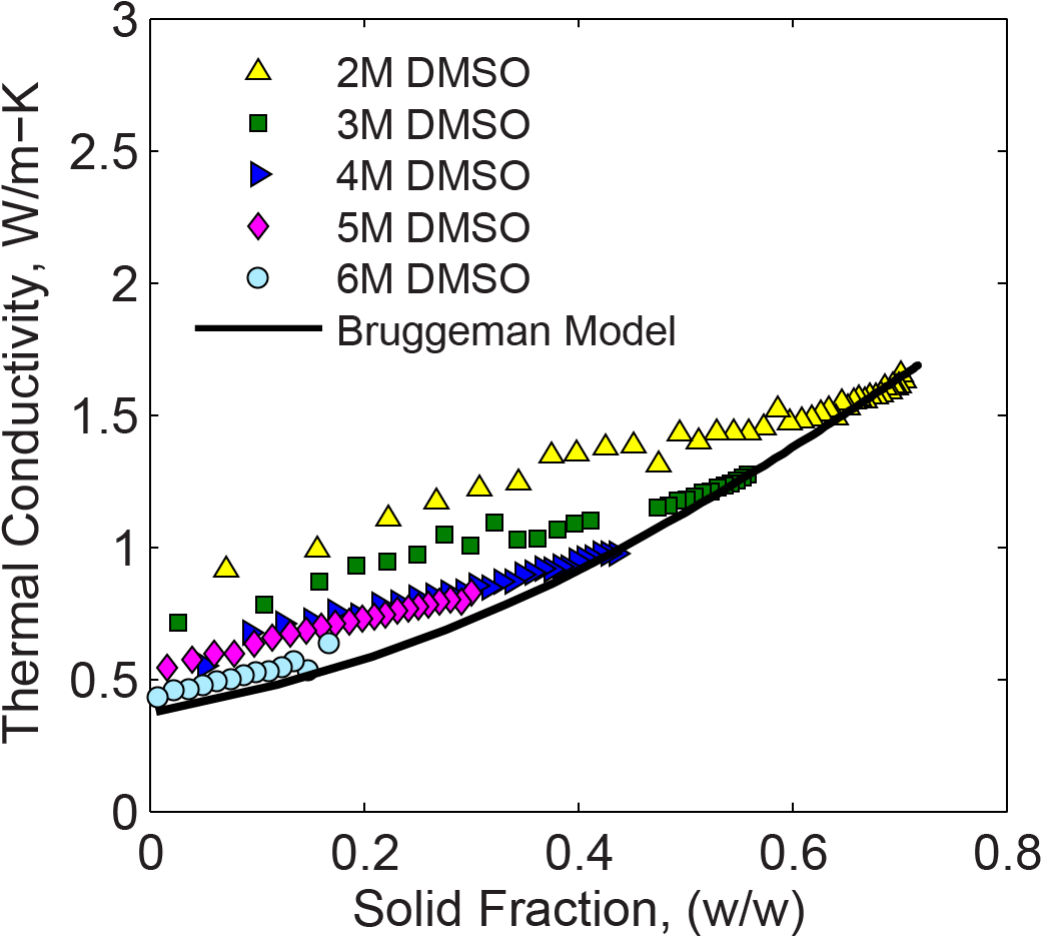
Thermal conductivity as a function of solid fraction for DMSO at various concentrations, where the Bruggeman model is calculated with Eq (7) for 6M DMSO.

Fig 9 displays an estimate of the effective thermal conductivity of DMSO based on the Bruggeman model, using the following assumptions: (i) component 1 is ice with thermal conductivity value of 2.5 W/m-°C, which is its actual value at -60°C; (ii) component 2 is 6M DMSO at the liquid state, with a thermal conductivity value of 0.43 W/m-°C, which is its actual value at -35°C; and, (iii) the thermal conductivity of each component is temperature independent. The above simplifying assumptions follow the observation that the thermal conductivity difference between pure water ice and liquid 6M DMSO within the range of interest is expected to be much more significant than the temperature dependency of each of those components individually over the same temperature range. Furthermore, while the results are more suitable to investigate phase transition in the 6M DMSO solution, the comparison with other solution concentrations is also insightful.

It can be seen from Fig 9 that the effective thermal conductivity increases with the increasing solid fraction, following a similar trend for the different concentrations. The maximum solid fraction for each concentration dataset is at the solidus temperature. The prediction of the Bruggeman model suggests that CPA undergoing phase transition may be first-order modeled as uniformly suspended crystals in a molten solution. The simplified model based on a 6M DMSO concentration and pure water ice properties appears to represent the same trend for other solution concentrations at higher solid fractions.

### Vitrified DMSO

No crystal formation was observed with the scanning cryomacroscope for DMSO concentrations of 7.05M or higher. For reference, Table 3 lists the liquidus and solidus temperatures for those higher-concentrations solutions extracted from a phase diagram [9], which reflects near-equilibrium conditions. Thermal conductivity for these concentrations monotonically decreases with decreasing temperature, which is typical of amorphous solids such as SiO_2_ and Poly(methyl-methacrylate) (PMMA). This behavior is attributed to the lack of long-range order typical of crystalline materials [10]. Similarly to crystalline DMSO, the thermal conductivity increases in value with the decreasing DMSO concentration, but with a much weaker dependency on concentration. A previously published thermal conductivity value of 0.32 W/m-°C for 7M DMSO at -20°C [12] is found to be in close agreement with a value of 0.34 ± 0.01 W/m-°C at -20.7 ± 0.8°C found in the current study.

**Table 3:** The liquidus temperature, *T*_*l*_, and the solidus temperature, *T*_*s*_, of solution concentrations observed to vitrify in this study, from a water-DMSO phase diagram [9].

DMSO concentration	*T*_*l*_, °C	*T*_*s*_, °C
7.05M	-54.2	-63.0
8M	-61.5	-63.0
9M	-67.3	-70.2
10M	-56.5	-72.8

Careful examination of the thermal conductivity data displayed in Fig 7 suggests minor latent heat effects for the 7.05M and 8M concentrations near the solidus temperature, which is associated with rewarming-phase crystallization (RPC). In general, RPC can either be the effect of devitrification—crystal formation during rewarming, or recrystallization—crystal growth from nuclei already formed during cooling. Either way, comparing the thermal conductivity trend and values below and above the solidus temperature suggests only minor crystallization effects.

To rationalize the observed temperature-dependent data for vitrified DMSO, it is compared with the Cahill-Pohl (C-P) model for thermal conductivity of amorphous solids [10]. The model is based on a scenario of an amorphous solid, where phonon-like collective vibrations of the atoms exist but scatter with very short mean free paths, equal to half of their wavelength. In contrast, the phonon mean free paths in crystals are hundreds to thousands of times their wavelength, leading to larger thermal conductivities as observed for lower molarity DMSO solutions. The C-P model has been validated against numerous amorphous materials including polymers and glasses. The C-P expression for thermal conductivity is:

$$k_{\min}={\left(\frac{\pi }{6}\right)}^{1/3}k_{B}n^{2/3}{\sum_{i}v_{i}\left(\frac{T}{\theta_{i}}\right)}^{2}\int_{0}^{\theta_{i}/T}\frac{x^{3}e^{x}}{{\left(e^{x}-1\right)}^{2}}dx\;;\;\theta_{i}=v_{i}\left(\text{h}/k_{B}\right){\left(6\pi^{2}n\right)}^{1/3}$$ (8)

where *i* refers to the polarization (e.g., transverse) of the phonon, ℏ is the reduced Planck’s constant, *k*_*B*_ is Boltzmann’s constant, and the model inputs *v*_i_ and *n* are the speed of sound and the number density of oscillators (typically one atom is an oscillator, but in some solids stiff bonds make clusters of atoms into oscillators), respectively.

Fig 7 displays the minimum thermal conductivity prediction for 10M DMSO based on the C-P model, using the following parameters: (i) the speed of sound is 1703 m/s for 10.12 M DMSO (used for all three polarizations) [14], and (ii) the number density of oscillators in 10M DMSO is 2.78×10^28^/m^3^, based on the number density of DMSO and H_2_O molecules, and the number of oscillators per molecule where stiff bonds are assumed between C-H and S = O in DMSO (each DMSO is three oscillators), and O-H in H_2_O (each H_2_O is one oscillator) in the temperature range of interest [20]. It can be seen from Fig 7 that the C-P model can be a useful tool in estimating the thermal conductivity in vitrified DMSO.

## Summary and Conclusions

The significance of the dependency of thermal conductivity on temperature has been established as an important consideration for heat transfer analyses of large systems undergoing cryopreservation [7]. The current study is aimed at quantifying this dependency in DMSO solutions, while taking into account two physical processes that affect the outcome of cryopreservation: crystallization and vitrification. This study utilizes the established hot-wire measurement technique, which is integrated for the first time into a recently developed device to visualize physical events during cryopreservation, termed the scanning cryomacroscope. The role of cryomacroscopy in this study is to verify the phase of state in the sample, while its thermal conductivity is continually measured along the thermal protocol. Also included in this study are reference measurements of thermal conductivity for pure water ice and glycerol, with comparable data available from the literature.

It is found in the current study that the thermal conductivity of the crystallized material varies significantly with the concentration, where samples in the concentration range of 2M and 6M DMSO where found to crystallize in the experimented thermal protocol. The thermal conductivity of the crystallized material is found to increase with the decreasing temperature, in the temperature range applicable to cryobiology (above -180°C in the current study). This behavior is expected to change at near-absolute zero temperatures. In contrast the thermal conductivity of vitrified DMSO solutions is not observed to be significantly dependent on concentration. The dependency of the thermal conductivity on temperature of the vitrified solution appears to follow the same trend from the liquid phase, which is to gradually decrease in thermal conductivity with the decreasing temperature. These opposing trends between the crystallized and vitrified material reach a tenfold difference at -180°C, which defines the lower boundary of the current experimental investigation. Such dramatic differences can drastically impact heat transfer during cryopreservation and their quantification is therefore critical to cryobiology.

## Supporting Information

S1 Appendix Uncertainty Analysis. (DOCX)
