# Supplementary material for: Large Thermal Conductivity Differences between the Crystalline and Vitrified States of DMSO with Applications to Cryopreservation
Source: PLoS One. 2015 May 18;10(5):e0125862. doi: 10.1371/journal.pone.0125862 (PMC4436132; doi:10.1371/journal.pone.0125862)
Supplement: S1 Appendix — (DOCX) [file pone.0125862.s001.docx]

# Appendix: Uncertainty Analysis

The uncertainty in thermal conductivity for a single measured value is estimated based on the uncertainty in the independent variables *δx_j_* used for thermal conductivity calculations:

| $\delta k_{sample}=\sqrt{\sum_{j} \left(\frac{\partial k_{sample}}{\partial x_{j}}\delta x_{j}\right)^{2}}$ | (A1) |
| --- | --- |

where *x_j_* stands for *I, R*_0_, *R*_ref_, *β*, and *L*. The thermal conductivity is expressed as:

| $k=\frac{I^{2}V_{i}\beta R_{0}}{4\pi LC}$ | (A2) |
| --- | --- |

where *C* represents that derivative:

| $C=\frac{\partial\left(\Delta V\right)}{\partial\left(\ln t\right)}$ | (A3) |
| --- | --- |

Special consideration is given to the estimating of *δC*, by taking the maximum change in slope of the corresponding best-fit approximation, as discussion in the context of Fig. 4.

Table A1 lists the values for the key parameters affecting the uncertainty in thermal conductivity measurements, which yield an overall uncertainty of ±0.03 W/m-°C according to Eq. (A1). From Fig. 8 it can be seen that the uncertainty in thermal conductivity measurements may be considered negligible for crystallized DMSO, but may get as high as 10% of the measured value for vitrified DMSO at -180°C. From the right column in Table A1 it can be seen that the most significant contributor to the overall uncertainty is *C*. Even if all other sources of uncertainty could be eliminated, the uncertainty in measurement due to the best-fitting of *C* would yield ±0.029 W/m-°C. Note that in reality, the uncertainty in *C* belongs to a wide range of values, and only the worst-case scenario is considered in the current analysis. Finally, note that the above analysis aims at the uncertainty in thermal conductivity for a single measured value. Additional variations between experimental runs and also between specimens are not accounted for in this analysis.

Table A1: Typical data used to estimate the overall uncertainty in a thermal conductivity measurement, based on the hot wire measurement technique and a sample of 7.05M DMSO.

| Parameter, *x*_j_ | Uncertainty, $\delta x_{j}$ | $\left(\frac{\partial k_{sample}}{\partial x_{j}}\delta x_{j}\right)^{2}$ |
| --- | --- | --- |
| Current, *I* | 0.09 mA | 2.43×10^-6^ |
| Measurement voltage, *V*_i_ | 6.96×10^-6^ … 2.75×10^-4^ V | 2.08×10^-8^ |
| Coefficient of thermal resistance, *β* | 5×10^-5^ °C^-1^ | 2.14×10^-5^ |
| Reference resistance, *R*_0_ | 5.7×10^-3^ Ω | 1.38×10^-8^ |
| Wire length, *L* | 1 mm | 2.44×10^-5^ |
| Variation in the ratio *C* | 1.76×10^-7^ … 1.72×10^-5^ V | 5.89×10^-7^ … 8.57×10^-4^ |
